# From Behavior of Water on Hydrophobic Graphene Surfaces to Ultra-Confinement of Water in Carbon Nanotubes

**DOI:** 10.3390/nano11020306

**Published:** 2021-01-25

**Authors:** Alia Mejri, Guillaume Herlem, Fabien Picaud

**Affiliations:** Laboratoire de Nanomédecine, Imagerie et Thérapeutiques, EA4662, UFR Sciences et Techniques, Centre Hospitalier Universitaire et Université de Bourgogne Franche Comté, 16 Route de Gray, 25030 Besançon, France; alia.mejri@univ-fcomte.fr (A.M.); guillaume.herlem@univ-fcomte.fr (G.H.)

**Keywords:** quantum simulations, carbon nanotube, graphene, functionalization, confinement

## Abstract

In recent years and with the achievement of nanotechnologies, the development of experiments based on carbon nanotubes has allowed to increase the ionic permeability and/or selectivity in nanodevices. However, this new technology opens the way to many questionable observations, to which theoretical work can answer using several approximations. One of them concerns the appearance of a negative charge on the carbon surface, when the latter is apparently neutral. Using first-principles density functional theory combined with molecular dynamics, we develop here several simulations on different systems in order to understand the reactivity of the carbon surface in low or ultra-high confinement. According to our calculations, there is high affinity of the carbon atom to the hydrogen ion in every situation, and to a lesser extent for the hydroxyl ion. The latter can only occur when the first hydrogen attack has been achieved. As a consequence, the functionalization of the carbon surface in the presence of an aqueous medium is activated by its protonation, then allowing the reactivity of the anion.

## 1. Introduction

Several curved and flat solid structures such as carbon (CNT) [1,2,3,4,5,6], boron nitrides (BNNT) and silicon carbide [7,8] nanotubes or surfaces [9,10] (graphene [11,12,13,14,15,16]) are interesting candidates for the design of synthetic nanofluidic platforms. The easy control of their diameter during the synthesis process can regulate inside liquid flow and transport of charges, opening up a wide field of applications in nanomedicine [17,18,19], biotechnology, desalination [20,21,22,23] membrane nanofiltration [24,25] nanofluidic devices for energy recovery and conversion [26,27,28,29,30,31,32] and water filtration [33]. CNTs are able to reproduce the biological properties of their counterparts, but with a less complex composition. For instance, they can notably exhibit chemical selectivity like certain natural nanochannels or transport different species. Many other different properties of bulk fluids could also be observed in such systems due to the surface effect.

Simulations and experiments with water confined inside carbon nanotubes can reveal unusual physical properties, especially for diffusion behavior and viscosity. These properties strongly depend on the geometrical characteristics of the CNT (tube diameter and chirality) and can directly affect water distribution inside the cage leading to unusual water performance in a confined space [34,35,36,37,38,39,40]. Several studies have shown for CNTs and BNNTs an ordered structure of water molecules essentially related to the metallicity and diameter of the tube. Pascal et al. reported that for armchair CNTs with increased diameters, water molecules present a bulk-like behavior when the CNT diameter is above 1.4 nm, while an ice-like water framework is characterized for CNT diameters ranging from 1.1 to 1.2 nm [41]. In a recent theoretical study, molecular dynamic simulations revealed that network formation in the form of a water chain occurred when molecules were successively arranged in CNT with diameters around 1.1 nm [39], which is in accordance with several previous studies [34,42,43,44]. Shayeganfar et al. reported, thanks to ab initio computations, that a water tube shape is observed when confined in CNTs and BNNTs. They also confirmed that this tendency of water arrangement depends on the diameter for both situations [45].

Otherwise, numerous experimental and theoretical studies carried out in recent years have shown that a significant surface charge in carbon and BN walls occurs in nanofluidic transport systems [10,46]. It has been established that this surface charge can be much higher for BNNT tubes than for CNTs. A plausible explanation for the appearance of this surface charge has remained puzzling. However, most of the available studies suggest that the adsorption of hydroxide ions on hydrophobic surfaces could explain this phenomenon.

Sirin et al. have shown in an experimental study that the high surface charge measured on a BNNT connecting two reservoirs could be related to the diameter of the tube as well as to the pH of the studied medium. The hypothesis of a chemical reactivity at the surface of BNNT was therefore underlined. On the basis of previous theoretical studies, it has been proposed that a site of “activated” boron could indeed cause the dissociation of water on the BN sheet [47,48]. Note that the carbon structures could also, both on a theoretical and experimental scale, show a particular ionic selectivity according to their diameter and their chirality [49,50], which could explain the specific charges of the carbon walls.

A good understanding of the mechanism governing the transport of fluid inside carbon-based materials, on a theoretical scale, would be an essential step in the development of new generation devices for a wide field of new industrial applications.

In fact, simulating the behavior of water molecules with respect to nanoporous solids is of great interest to investigate promising materials for smart nanofluidic systems under electric bias [51,52,53,54]. Consequently, recourse to computational methods would allow a realistic approach to be established by reproducing an electrochemical system in which the electrolytes are in contact with a solid polarized surface under the effect of an external uniform electric field [3,55,56].

Otani and O. Sugino [57] have developed since 2006 a novel computational scheme that makes it possible to apply an electric bias to the system constituting a slab as occurring with an electrode and an electrolyte solution. The slab represents a bounded polarized or charged interface between two semi-infinite media having a dielectric constant. The method is then called “Effective Screening Medium”. The boundary conditions are given to a model unit cell by solving the Poisson equation allowing the creation of an infinite slab.

The Effective Screening Medium (ESM) method allows, through the coupling of DFT and molecular dynamics, a rigorous study of electrochemical systems. In the present study, two solid structures were tested against dissociated and undissociated water: the zigzag carbon nanotube and the graphene monolayer. Various quantities were then extracted from this study, in particular the adsorption energy of water on the solid surface, the radial distribution density of the confined water as well as the relevant structural observations.

## 2. Materials and Methods

First-principle density functional theory (DFT) calculations were used to investigate the interaction of a dissociated and undissociated water molecule with graphene and the carbon nanotube. The geometry optimization was performed through the “Open source package for Material eXplorer code” (OpenMX) using a combination of molecular dynamics, density functional theory and generalized gradient approximation for the exchange-correlation energy proposed by Perdew, Burke and Ernzerhof (GGA-PBE). Pseudopotentials and wave functions have also been implemented to reduce the calculation cost. Structural and energetical properties were investigated on the studied systems such as adsorption energy, ground state geometries of system components and electronic density of states (DOS). Differences in charge density calculations were also performed by OpenMX code for the adsorption of dissociated water molecules on CNT and graphene structures. This implies a more rigorous understanding of the spin (charges) density redistribution induced by the interaction of water entities with carbon structures. Although it is frequently used in the description of the electronic structure of a system, DFT based on the generalized gradient approximation has certain limitations, in particular for the modeling of chemical reactions [58] and the estimation of gas-phase energy barriers [59]. DFT-GGA may also not work well for many molecule–metal surface reactions and for van der Waals adsorption on surfaces [60]. Ab initio molecular dynamics based on density functional theory are more reliable and accurate in describing molecule–surface interaction, reaction pathways [61], adsorbate diffusion [62,63] and energy exchange as it permits surface-atom movement and also includes the temperature effect [64].

The total energy scf convergence criterion for the self-consistent electronic minimization is set to 10^−6^ Hartree/supercell (i.e., 0.27 × 10^−8^ eV/Å^3^). Pseudo-atomic orbitals (PAOs) centered on atomic sites were used as basis sets. The basis sets for C, O, Cl, B and N were taken as “s2p2d1”, while those for Na atoms were “s2p2”. The k points are generated according to the Monkhorst–Pack method and were set to 3 × 3 × 1. The mesh cut-off energy value was set to 170 Ry (i.e., 2313 eV). Otherwise, a large 34 Å vacuum is built into the cell along the z axis to avoid overlapping periodic cells. Note that the van der Waals corrections were not taken into account in our calculations. The choice of empirical parameters dedicated to the modeling of these corrections in DFT could increase the main source of uncertainty in our calculation. This would lead to shifts in energy, which will always be submitted to discussion [65].

The adsorption energy (Equation (1)) is estimated based on a difference between the total energy of the complex tube CNT (and graphene) + adsorbate system and the individual tube (and graphene) and gas phase free molecule system.

Eads H^+^/HO^−^ = Etot (H^+^/HO^−^ads_surface) − E(H^+^/HO^−^des_surface) (1)

For all the simulations, molecular dynamics calculations were carried out in the NVT ensemble at 300 K. The velocities of the atoms were scaled every 20 MD steps, and time step was 1 fs. All simulations were run for 2000 fs.

Monolayer graphene is made of 32 atoms and adopts an armchair chirality (1,1) with honeycomb structure and semi-metallic properties. The monolayers of each system are separated by a 34 Å vacuum to avoid any interaction between the periodic images.

**Figure 1:**
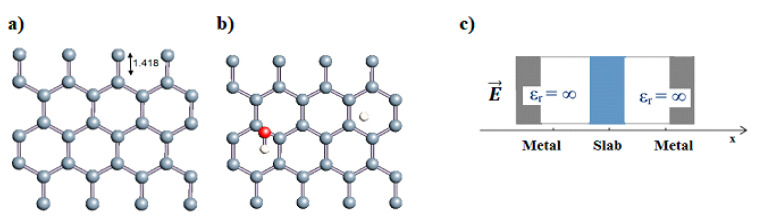
(**a**,**b**) Graphene and dissociated water + graphene system. (**c**) ESM method model.

Carbon nanotubes were also studied with a confined water molecule and the same vacuum exclusive region, as previously mentioned. For all the structures, two situations were investigated: a first case with an undissociated water molecule and a second one with a dissociated water molecule. In each situation, the cases without field and with field application were also explored. The electric field, when applied, was along the x axis of the elementary cell presented in Figure 1c. The studied slabs (CNT and graphene layer) and ESMs were placed parallel to the y-z plane. The electric field was therefore applied perpendicularly to the tube axis and to the graphene plane. The effective screening media (ESMs) were placed at the cell boundaries conforming to Figure 1c. Note also that the origin of the x-axis was set at the cell boundary.

## 3. Results

### 3.1. Water Molecule Interaction with Graphene Walls

Graphene has become a key component in the development of graphitic nanoslits for the transport of water and ions [66,67,68]. However, there is still an important lack of theoretical studies that analyze the behavior of water with respect to this material since many experimental observations are still interpreted as coming from the apparition of a surface charge. The origin of the latter needs more profound theoretical insight to understand its appearance. Hence, it seemed relevant to investigate more closely the behavior of a dissociated water molecule near a single graphene sheet. A uniform electric field was applied to the system to model the influence of the potential drop used in current–voltage measurements. Figure 1 shows the studied system and summarizes the ESM method model used in these calculations.

The same calculations were also performed for an undissociated water molecule; the applied field did not cause the spontaneous dissociation of the water molecule, even for high intensities.

As shown in Table 1, which summarizes all the adsorption energies of H^+^ and HO^−^ on the graphene surface due to the most important events occurring during the simulation, the adsorption states of H^+^ and HO^−^ were all negative, indicating favorable adsorption in each case. The first adsorption energy of each entity is called E_ads_. H^+^ and Eads HO^−^.

In the three 2000 fs simulations, the adsorption of the H^+^ was noted at fast times. For fields equal to 0 eV and −5 eV, HO^−^ adsorption was not observed. A very high field intensity alone allows the adsorption of HO^−^ to occur. Note that the values of the electric field should be transformed to be expressed in a usual unit. For each calculation, we had to transform U (in eV) to U (in V/Å), by dividing the initial value by the length of the cell box (i.e., 34 Å). As a consequence, 1 eV was equal to 4.7 × 10^−21^ V/Å)

The hydrogen adsorption energies were in agreement with the theoretical calculations observed in the literature, which ranged from −0.81 [69] for the PBE method to −0.67 in LSDA [70].

The adsorption energy of HO^−^ was not favored in the first two situations, when the electric field value was weak. It can only occur with a strong field but presents a value which remains in agreement with the literature for this type of system. Note that the adsorption of HO^−^ is possible only after a first adsorption of H^+^, which allows the imbalance of the charge carriers in the planar surface. This has already been observed in recent data since the HO^−^ adsorption on graphene was never chemical, and it leads to small interaction energies with carbon atom.

**Figure 2:**
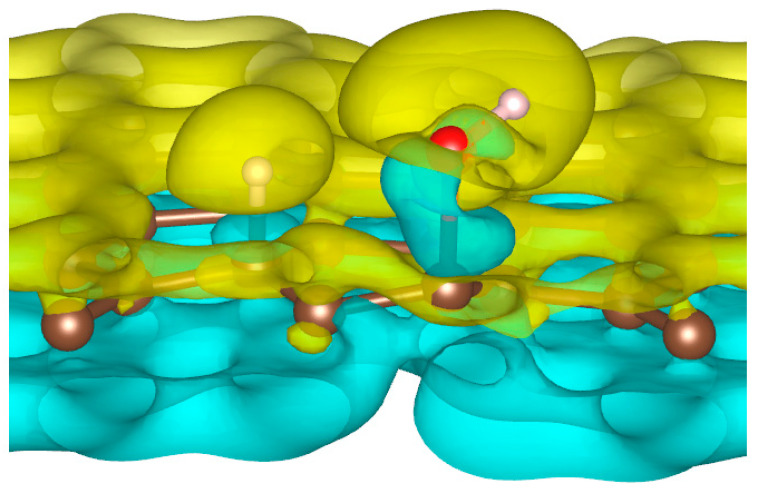
Charge density distribution in the case of dissociated water molecule adsorption on graphene at −50 eV electric field. Yellow and blue lobes represent, respectively, the positively and negatively charged areas.

To better understand the ability of hydrogen or hydroxyl ions to interact with the graphene sheet, we represent in Figure 2 the charge density distribution differences for a dissociated water molecule near a graphene sheet.

As shown in Figure 2, the surface polarization generated by the effect of the electric field creates negative and positive charges on the carbon atoms of graphene. This polarization allows the H^+^ ion to be adsorbed on the carbon atoms, which has a negative surface layer. Indeed, H^+^ is forced to translate in the field direction, as do the partial charges on the graphene surface. This induces a favorable adsorption of H^+^ at the first step of the simulation. Once H^+^ is bonded to a carbon atom, it locally modifies the density of charge repartition. Without such changes, HO^−^ could never be adsorbed on the graphene surface. The presence of the cation thus allows HO^−^ to be attracted by the graphene surface spontaneously.

#### Salt Effect

The role of salt in water dynamics is necessary to complete the simulated system and get closer to the experimental conditions. The dissociated sodium chloride (Na^+^, Cl^−^) was thus added to the previous system.

The behavior of water and salt with respect to graphene at different field strengths is given in Table 2.

In all simulations, the reformation of the water molecules of H^+^ and HO^−^ in solution was observed at relatively short times for all field intensities. However, for weak field intensities, short-lived interactions of H^+^ with the carbon surface are possible but are not really relevant. There was no real HO^−^ and H^+^ adsorption phenomena on the graphene surface in the presence of salt in these simulations. Note that during simulations, the reformation of NaCl was observed near the graphene surface in our electrochemical ESM cell. There is, thus, no possibility for salt ions to be kept by the graphene surface.

### 3.2. Undissociated Water Molecule inside the Carbon Nanotube

**Figure 3:**
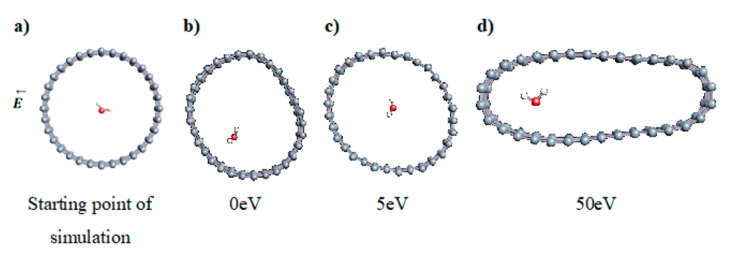
Electrical polarization effect on a water@tube system. (**a**) Initial configuration. (**b**–**d**) final configuration for E = 0 eV (5 eV and 50 eV, respectively)

The role of confinement at the nanometric scale on the possibility to charge a carbon wall was then studied. Indeed, it has been established in previous experimental and theoretical studies [71,72,73] that water dissociation can occur under the effect of an electric field. Furthermore, studies of the water behavior in an ultra-confined environment have not excluded the possibility of its dissociation [74,75]. This dissociation can be highly favored in a confined space, in fact, Muñoz-Santiburcio et al. have shown that confinement greatly improves the self-dissociation process of water. This result is consistent with another study conducted by Sirkin et al. who used QM/MM molecular dynamics to compute the energy without water dissociation in a single-walled carbon nanotube 8.1 Å in diameter. They hypothesized that it seems plausible, under the effect of nanometric confinement, to see an increase in the self-dissociation constant due to the increase in the permittivity of the confined fluid [75]. We first modelized a (16,0) single-walled carbon nanotube with diameter of 1.35 nm where a water molecule was introduced into the confined inner space of the carbon cage. Several situations have been achieved by increasing the field intensity (Figure 3).

Despite the importance of the applied field intensities that strongly impact the geometry of the carbon nanotube, we did not observe dissociation of a confined water molecule. There was a deformation of the nanotube until it was crushed and formed an elongated shape in the transverse direction (Figure 3d). Whatever the deformation, the molecule diffused inside the internal volume of the CNT, exploring different atomic positions, but keeping its distance from carbon wall due to hydrophobic interaction [76]. Note that no form of physical or chemical adsorption of the water molecule was noted on the carbon surface.

#### 3.2.1. Dissociated Water Molecule Inside CNT

No dissociation of the molecule has been observed in our previous simulations. The next step of our calculations deals with the simulation of a dissociated water molecule inside the carbon cage. In this case, we directly studied the possibility of hydronium and hydroxyl ion adsorption resulting from this dissociation and quantified it in terms of adsorption energy. Several simulations were undertaken for a dissociated water molecule confined inside the carbon nanotube (16,0). The main results are shown in Table 3 and Table 4.

We first noted that the H^+^ adsorption was possible spontaneously without an external contribution of an electric field, as seen for the first simulation at 0 eV field intensity. In addition, our calculations show that the adsorption of H^+^ always preceded that of HO^−^ regardless of the intensity of the applied field. Note here that hydrogen adsorption was favored rapidly and did not depend on the deformation of the carbon cage under the electric field intensity. The rapid process leading to the hydrogenation of a carbon was observed before the strong modification of the carbon geometry. On the contrary, the formation of a water molecule (observed for E = 10 eV) or the adsorption of hydroxyl was only possible when H^+^ was chemisorbed and the carbon surface was deformed under an increasing electric field intensity, as observed previously.

The last two simulations gathered in Table 3 (performed at 15 eV and 25 eV) recall the case of graphene for a dissociated water molecule. In fact, HO^−^ adsorption took place at later times in the simulation but especially at high field intensities and for significant carbon deformation. Note that the adsorption of HO^−^ (or the reformation of water molecule) was not observed due to the simulation time, which was stopped equally for each calculation. We reported in Table 4 the different adsorption energies obtained when hydrogen and/or hydroxyl ions are adsorbed on the carbon wall.

The energies calculated for H^+^ adsorption on the inner surface of the carbon cage were on the order of −4 eV. These clearly show that the adsorptions observed were chemisorptions, explaining the difficulty for hydroxyl ions to interact with hydrogen once chemisorbed. These values are in agreement with others found in the literature, which are around −3 eV [77]. Note also that for each modification of the carbon surface by the hydrogen chemisorption, we observed a modification of the carbon hybridation, which could be apparent to a sp^3^ mode. The hydroxyl ion interacted with the carbon surface with a higher energy, which is comparable to those obtained in the literature [78].

#### 3.2.2. Differences in Charge Density Distribution for the Dissociated Water Molecule Inside CNT

**Figure 4:**
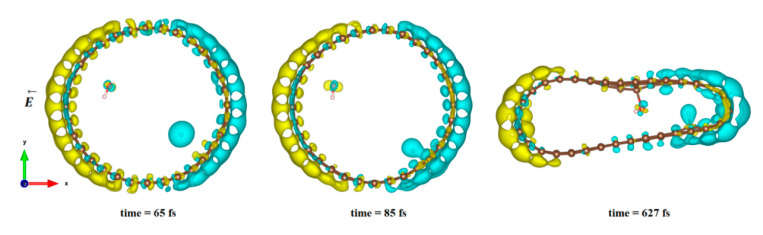
Difference in charge density distribution of the dissociated water molecule inside the CNT under 25 eV electric field. The yellow and blue lobes represent the positively and negatively charged areas, respectively.

In Figure 4, we plot the modification of the atomic charge density when applying a high electric field intensity (25 eV). The positive and negative differences in the total charge densities are colored in yellow and blue, respectively. As can be seen in Figure 4, polarization of the surface is responsible for delocalization of the electrons and, therefore, for the creation of an electron deficit on certain areas of the internal surface of the tube and an accumulation of electrons in other areas. As a consequence, the hydrogen ion will be more sensitive to the surface zone where electrons are present, while the hydroxyl remains close to the oppositely charged surface part while the CNT is slightly deformed. However, even in this large field intensity, the time necessary to obtain the hydroxyl binding to the carbon surface was quite large (627 fs), while the hydrogen ion attached faster to the surface (85 fs compared to 110 fs at least). The final adsorption of hydroxyl was observed on the flatter surface of the deformed CNT, where the strain appeared to be the least. Indeed, it has been shown in previous studies that the tensile strain on a single sheet of graphene can influence the interaction of the adsorbents but also make possible the modification of its mechanical and physical properties [79,80,81,82].

#### 3.2.3. Effect of Adding Water Molecules on the Adsorption Steps

To go further in our study, we complicated the previous system by adding an additional water molecule and let the system evolve to see its effect on the adsorption steps. Several simulations were performed by varying the intensity of the applied field. A domain of intensities ranging from 0 to 30 eV was scanned. Table 5 illustrates all the simulations carried out for this system containing one dissociated and one undissociated water molecule inside the carbon nanotube (16,0).

As shown in Table 5, almost the same behavior was detected in all the simulations, even at high field strengths. The phenomena of H^+^ and HO^−^ adsorption occurred at practically simultaneous instants with a very slight advance of HO^−^ adsorption of a few fs over the H^+^ adsorption, compared to the previous system. This first HO^−^ adsorption, before any other, was the main difference obtained in this system, which has never been observed previously. However, it is not very durable because the entity was desorbed in all cases after 40 fs of existence, depicting a very low adsorption energy with the carbon atom. On the other hand, H^+^ remained adsorbed until the end of the simulation in all situations, as observed previously. We can therefore wonder about the role of HO^−^ on the H^+^ adsorption in this case. It can either be the main factor having improved the association of hydrogen with carbon by the modification of the electronic structure of the cage or, simply, be the random consequence of the hydroxyl position compared to the hydrogen position. Note that no dissociation of the water molecule was observed during the simulation.

The adsorption energies were calculated. Results are reported in Table 6. Due to very fast hydroxyl adsorption events, we were not able to estimate the adsorption energy for the HO^−^ ion. However, as seen in Table 6, the hydrogen adsorption energy was equal to −4.4 eV, as obtained previously (Table 4), for the system where no water molecule was present. The role of the water molecule added to the hydrogen plus hydroxyl ion seems to play a minor role in the reactivity of the carbon surface.

#### 3.2.4. Salt Effect on Adsorption Phenomena

In order to evaluate the effect of the ions on the adsorption of dissociated water inside carbon nanotube, we added to the dissociated H_2_O@CNT system a salt composed of a unique Na^+^ ion and its Cl^−^ counterion. The different adsorption events as a function of the increase in field intensity are summarized in Table 7. For intensities between 5 and 20 eV, the H^+^ adsorption first occurred at around 80 fs followed by the rapid reformation of the water molecule. At a field of 25 eV, HO^−^ adsorption occurred first, at about 385 fs, and the entity remained adsorbed for 200 fs. Note that CNT was much less deformed under the action of an intense electric field when it contained more molecules, and no dissociation of the water molecule was observed once formed.

As for other systems, we estimate the H^+^ and HO^−^ adsorption energies in Table 8. We observe that the adsorption of H^+^ was less favorable in this case (−4 eV at best), while the adsorption of HO^−^ in the very high electric field intensity was on the same order as that of H^+^. The rapid desorption of HO^−^ cannot explain this result, but the presence of Na^+^ allows it. Indeed, we observe an important role played by the salt, which is alternatively attracted by the hydrogen or hydroxyl ions to form another strong acidic or basic component.

#### 3.2.5. Several Water Molecules Inside (16,0) Carbon Nanotube

In order to get closer to biological conditions, a dissociated water molecule system immersed in several water molecules was simulated by varying the intensity of the applied electric field. The density of water was calculated to be 1 in order to reproduce a bulk-like water media. After 2000 fs simulations we observed in all cases a rapid formation of water molecules (in 17 fs).

In order to check the conformation of the confined water and to see if possibly a phase change occurred (Table 9), we calculated the radial distribution density of the water in the various situations studied. The calculated values are entered because the water at the end of the simulation keeps the structure of the liquid phase and summarized in Table 10. For each case, the first peak localized near 2.7 Å. This value corroborates the organization of the water molecule in liquid form since the experimental value for liquid water is 2.88 Å.

Note that during the simulation, while no adsorption was observed on the carbon surface, the formation of successive hydronium ions inside the water bulk and proton jump have been affected via the so-called Grotthuss mechanism.

### 3.3. Change in Hybridization of the Adsorption Site

We have found by comparing the two carbon structures that the adsorption of HO^−^ on carbon nanotubes was much more favorable than on a graphene monolayer and took place at lower field intensities. This probably is due to the higher surface charge of the carbon nanotube and its coiled structure.

**Figure 5:**
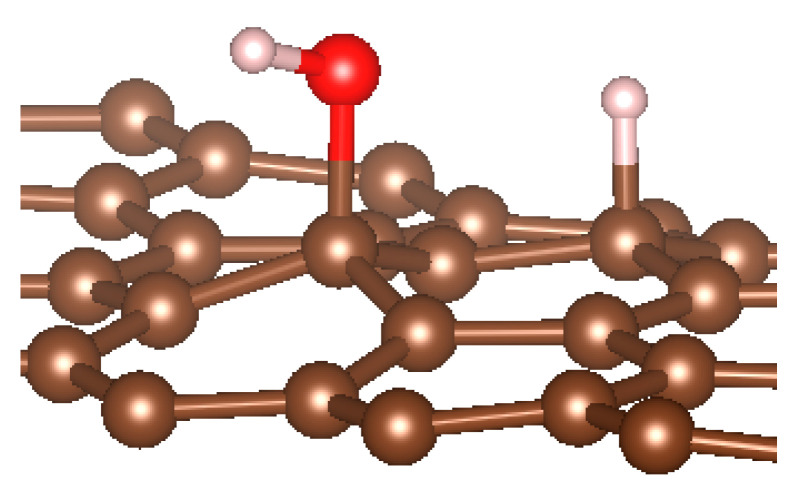
sp^3^ hybridization of the adsorption site for graphene material.

We noted here for the carbon structures that a change of conformation was observed for the carbon atom at the adsorption site (see Figure 5). Indeed, as established by previous studies, the adsorption of an entity on a graphene surface or on the internal or external surface of a single-walled carbon nanotube modified the initially hybridized adsorption site sp^2^ (planar structure). Due to adsorption, it deviated from its original state towards sp^3^ type hybridization. It is then the center of a regular tetrahedron defined by three adjacent carbon atoms and the adsorbed entity. This strong local deformation causes a change in the bond angles of the original sp^2^ hybridization, (CCC) = 120° to (CCC) = 112°, respectively, for CNT and graphene.

Note also that the observed bond lengths were C-H_graphene_ = 1.1 Å; C-H_CNT_ = 1.12 Å; C-O_graphene_ = 1.52; C-O_CNT_ = 1.496Å, which are very characteristic of single bonds for each of the studied structures. The puckering of the carbon atom under the adsorbed hydrogen atom led to an increase in its sp^3^ character [10,11,15,77,82,83,84,85,86,87]. There was also a stretching of the C−C bonds associated with this carbon atom. They were approximately 0.3 Å elongated from the original C−C bond length in pure structures. Casolo et al. quantified the electronic rearrangement of the carbon atom by a high-energy barrier of 0.2 eV [10,11,15,77,82,83,84,85,86,87,88].

## 4. Conclusions

In this work, we have studied, through DFT-MD calculations, the analysis of flat (graphene) or curveted (CNT) carbon surface reactivities to proton and hydroxyl ions or hydroxyl ions, mixed or not with other entities, upon the presence of an electric field or not at the molecular scale. From all of our studies, we have shown a very strong affinity of the carbon wall, whatever its curvature, for the proton, with a notable modification of the hybridation of carbon atom as observed in recent literature [89,90,91,92,93]. The adsorption energy obtained in each case was about −4 eV, in agreement with the literature. On the contrary, we can note that no specific adsorption preferences were characterized for the HO^−^ ion. Some punctual observations of hydroxyl interaction with the carbon surface were obtained, but mainly after a first functionalization of the carbon by the hydrogen in the presence and absence of an electric field. However, the higher the electric field intensity, the faster the proton chemisorption rate. For graphene, the presence of a dissociated salt (NaCl) with water led to desorption of ions, while HO^−^ can adsorb first as observed in CNT charged with NaCl. This asymmetry of ion adsorptions occurs on flat and curveted carbonaceous surfaces but can be drastically affected by an external electric field, while being pH-dependent in water.

## Figures and Tables

**Table 1:** Adsorption states and energies of dissociated water molecules on the graphene monolayer.

U(eV)	0	−5	−50
Figure	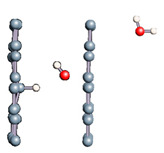	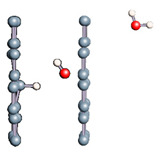	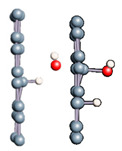
Observation	H^+^ adsorption at 79 fs. H_2_O formation at 365 fs.	H^+^ adsorption at 63 fs. H_2_O formation at 365 fs.	H^+^ adsorption at 40 fs. HO^−^ adsorption at 906 fs.
Eads. H^+^ (eV)	−0.9	−1.3	−1.5
Eads. HO^−^ (eV)	-	-	−0.6

**Table 2:** Behavior of the dissociated water molecule near the graphene layer in the presence of salt under electric bias.

U(eV)	Important Events in Simulation	Observations
0	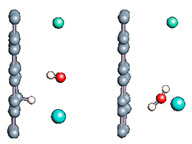	H^+^ adsorption at 135 fs. H_2_O formation at 292 fs.
5	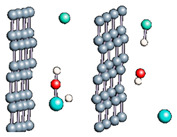	NaOH formation at 100 fs. HCl Formation at 242 fs. H_2_O formation at 815 fs.
−5	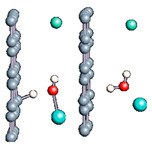	NaOH formation at 110 fs. H^+^ adsorption at 120 fs. H_2_O formation at 240 fs.
50	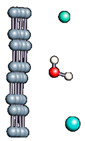	H_2_O formation at 220 fs.
−50	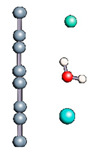	H_2_O formation at 101 fs. NaCl formation at 175 fs.

**Table 3:** Water molecule dissociated inside (16,0) CNT under electric bias.

U(eV)	Important Events in Simulation	Observations
0	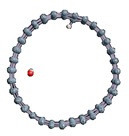	H^+^ adsorption at 140 fs.
1	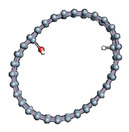	H^+^ adsorption at 112 fs. HO^−^ adsorption at 212 fs.
5	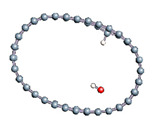	H^+^ adsorption at 113 fs.
10	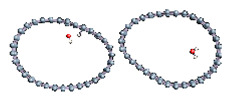	H^+^ adsorption at 110 fs. H_2_O formation at 456 fs.
15	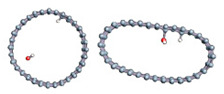	H^+^ adsorption at 95 fs. HO^−^ adsorption at 470 fs.
25	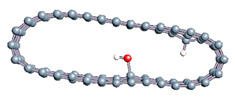	H^+^ adsorption at 104 fs. HO^−^ adsorption at 589 fs.

**Table 4:** (H^+^, HO^−^) Adsorption energies inside (16,0) CNT.

U(eV)	H^+^ Ads. Energy (eV)	HO^−^ Ads. Energy (eV)
0	−4.1	-
1	−4.2	−0.3
5	−4.0	-
10	−4.6	-
15	−4.6	−0.06
20	−4.3	-
25	−4.2	−0.3

**Table 5:** Dissociated and undissociated water molecules inside (16,0) CNT under electric bias.

Field Intensity (eV)	Important Events	Observation
1	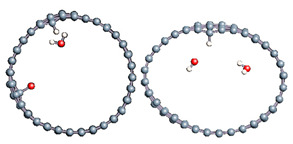	HO^−^ adsorption at 134 fs. H^+^ adsorption at 150 fs. HO^−^ desorption at 179 fs.
10	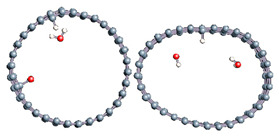	HO^−^ adsorption at 135 fs. H^+^ adsorption at 137 fs. HO^−^ desorption at 173 fs.
15	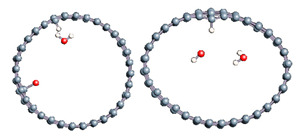	HO^−^ adsorption at 133 fs. H^+^ adsorption at 137 fs. HO^−^ desorption at 167 fs.
20	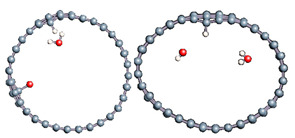	HO^−^ adsorption at 133 fs. H^+^ adsorption at 138 fs. HO^−^ desorption at 173 fs.
30	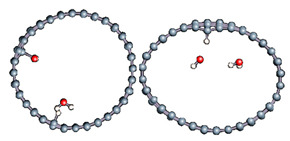	HO^−^ adsorption at 133 fs. H^+^ adsorption at 136 fs. HO^−^ desorption at 177 fs.

**Table 6:** H^+^ Adsorption energies inside (16,0) CNT.

U(eV)	H^+^ Ads. Energy (eV)
1	−4.413
10	−4.408
15	−4.426
20	−4.409
30	−4.508

**Table 7:** Dissociated water molecules inside (16,0) CNT under electric bias in the presence of a salt.

Field Intensity (eV)	Important Events in Simulation	Observations
0	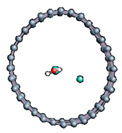	H_2_O is formed at 250 fs.
5	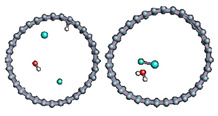	H^+^ adsorbed at 75 fs. H_2_O is formed at 551 fs.
10	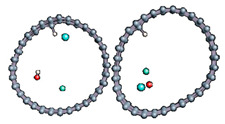	H^+^ is adsorbed at 90 fs and HO^−^ remains free until the end of the simulation
20	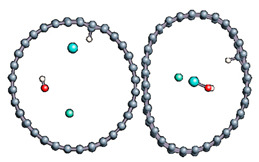	H^+^ is adsorbed at 83 fs and HO^−^ remains free until the end of the simulation. NaOH formation.
25	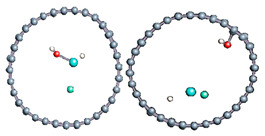	NaOH formation at 268 fs. HO^−^ adsorption at 385 fs. HO^−^ desorption at 556 fs.

**Table 8:** H^+^ Adsorption energies inside (16,0) CNT in the presence of salt.

U(eV)	H^+^ Ads. Energy (eV)	H^+^ Ads. Duration (fs)	HO^−^ Ads. Energy (eV)	HO^−^ Ads. Duration (fs)
0	-	-	-	-
5	3.288	476	-	-
10	−3.449	1910	-	-
20	−4.087	1917	-	-
25	-	-	−3.211	171

**Table 9:** Distribution of water inside the (16,0) carbon nanotube.

Field Intensity (eV)	0	10	25	50
Water distribution	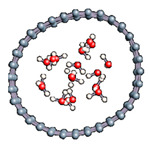	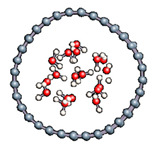	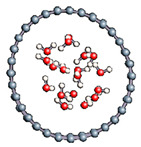	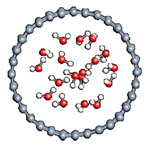
First maximum position (Å)	2.66	2.73	2.75	2.75

**Table 10:** First peak position in the radial distribution function of confined water.

U(eV)	First Maximum Position (Å)
0	2.658
10	2.73
25	2.754
50	2.75
Experimental value for liquid water	g_OO1_ = 2.88

## Data Availability

Data available on demand.
